# 

**Published:** 1884-06-20

**Authors:** 


### *READ! READ!!*

☞ Many of our subscribers are in arrears. Friends, the aggregate of these small amounts is very important to us. Please let us hear from you at ONCE.

R. C. WORD,
Managing Editor.

### RECEIPTED.

1884.—Drs. G. L. Landers; John A. Nelms; L. G. Hardman; H. Alison; L. N. Hyten; P. George; R. L. Tatum; James Pointdexter; Thos. A. Killes; Mark Williams; John L. Johnston.
1883.—J. Covington; R. B. Norton; Sam'l. Thompson; E. D. Edwards.
1885.—J. S. Horsey.

